# Effects of Ketogenic Diet (KD) on Metabolic, Endocrine, and Reproductive Outcomes in Overweight/Obese Women With Polycystic Ovary Syndrome (PCOS): A Systematic Review

**DOI:** 10.7759/cureus.103615

**Published:** 2026-02-14

**Authors:** Arpita Misra Diha, Md Sadman Sakib Uddin, Kamarun Munira Anha, Fraol Dereje Bekele, Jannatul Naima, Abdulaziz Ahmed Salem Al-Khulaifi, Amina Abdul Nassar, Md Sajidul Huq

**Affiliations:** 1 Department of Food and Nutrition, Government College of Applied Human Science, Dhaka, BGD; 2 Department of Nutrition and Food Engineering, Daffodil International University, Dhaka, BGD; 3 Department of Public Health, Johns Hopkins Bloomberg School of Public Health, Baltimore, USA; 4 Department of Medicine, Shaheed Monsur Ali Medical College, Dhaka, BGD; 5 Department of Medicine, Mendero Medical Center, Cebu, PHL; 6 Department of Plastic and Reconstructive Surgery, Aster MIMS Hospital, Kannur, IND; 7 Department of Public Health, Deep Eye Care Foundation, Rangpur, BGD

**Keywords:** insulin resistance, ketogenic diet, overweight/obese, polycystic ovary syndrome, reproductive health, weight loss

## Abstract

Polycystic ovary syndrome (PCOS) is a common endocrine disorder frequently complicated by obesity, insulin resistance, and reproductive dysfunction. The ketogenic diet (KD), characterized by very low carbohydrate intake, has emerged as a potential intervention, but its comprehensive short-term effects in this population require synthesis. This systematic review evaluates the impact of KD on metabolic, hormonal, and reproductive outcomes in overweight and obese women with PCOS. Following Preferred Reporting Items for Systematic Reviews and Meta-Analyses (PRISMA) 2020 guidelines, a systematic search of multiple databases was conducted for studies published up to 2024. Randomized controlled trials (RCTs), comparative studies, and prospective cohorts assessing a KD (≤50 g carbohydrates/day) over 6-24 weeks in overweight/obese women with PCOS were included. Fourteen studies involving 643 participants (n = 240 from RCTs; n = 403 from observational or single-arm studies) were analyzed. Most studies reported significant weight loss (7-13% of baseline weight) and reduced visceral adiposity. Marked improvements were observed in insulin sensitivity, with reductions in fasting insulin, glucose, and homeostatic model assessment of insulin resistance (HOMA-IR). Hormonal profiles improved, evidenced by decreased total and free testosterone, increased sex hormone-binding globulin (SHBG), and normalization of the LH/FSH ratio. Reproductive benefits, including restored menstrual regularity and increased ovulation rates, were reported, although pregnancy outcomes were derived from small, uncontrolled cohorts and should be interpreted cautiously. Lipid profile changes were mixed but generally favorable, and adverse events were typically mild, though adherence challenges were noted. Low-to-moderate certainty evidence suggests that KD demonstrates significant short-term efficacy for improving weight, metabolic parameters, androgen excess, and reproductive function in overweight/obese women with PCOS. However, the evidence is limited by study heterogeneity, short durations, and varied protocols. These findings support KD as a promising therapeutic option, but larger, long-term RCTs are needed to establish its safety, sustainability, and optimal implementation within PCOS management.

## Introduction and background

Polycystic ovary syndrome (PCOS) is a prevalent endocrine disorder with a worldwide prevalence estimated to be 10-15% in reproductive-aged women, depending on the diagnostic criteria used [1]. It can be described as a hormonal imbalance, which is mainly caused by the ovaries producing high amounts of androgens [2]. This leads to abnormal menstrual cycles in people with PCOS, missed ovulation, and unpredictable ovulation [3]. PCOS is a common cause of infertility in women and is linked with higher risks of other diseases, including central obesity, insulin resistance, hyperinsulinemia, type 2 diabetes mellitus, and dyslipidemia [4,5].

A high percentage of women with PCOS are obese, with the research indicating estimates of 38-88% of women based on the population and clinical environment under investigation [6,7]. The adipose tissue, especially visceral fat, is an active metabolic and endocrine gland, and obesity is a factor that worsens metabolic and reproductive defects linked to PCOS [8]. The surplus adiposity facilitates insulin resistance and compensatory hyperinsulinism, which inhibits hepatic synthesis of sex hormone-binding globulin (SHBG) and stimulates ovarian androgen synthesis to cause a hyperandrogenic condition [9,10]. Visceral obesity is an important risk factor that increases the likelihood of impaired glucose tolerance, type 2 diabetes, metabolic syndrome, and cardiovascular morbidity, and these risks are especially high in women with PCOS [11].

Weight loss remains central to the PCOS treatment and even small weight losses improve ovulation, insulin resistance, and androgen status [12]. Nevertheless, the conventional dieting approaches, such as low-calorie and low-glycemic index diets, are frequently not followed, which undermines long-term performance. This has opposed other nutritional interventions such as very low-carbohydrate ketogenic diets (KD), which have proved to have a positive effect on metabolic parameters and weight control.

KD is a very low-carbohydrate, adequate-protein, high-fat dietary pattern, generally with less than 50 g of carbohydrates per day, or 5-10% of the total energy consumption [13]. Although the exact proportions of macronutrients can be varied, the diet is usually made of 60-80% of calories in the form of fat and 10-30% in the form of protein [14]. These proportions can be adjusted depending on individual therapeutic goals, such as weight loss, glycemic control, or management of metabolic or neurological conditions. The objective is to induce a metabolic state of ketosis, where the body shifts its primary fuel source from glucose to fatty acids and ketone bodies derived from dietary fat and adipose stores [15].

Despite the symptoms of PCOS being reduced by weight management, the conventional low-calorie/low-glycemic index diets are usually characterized by low adherence. KD has the potential to lose weight, improve insulin sensitivity, and regulate hormones in overweight/obese women with PCOS, but the existing studies feature small sample sizes, heterogeneous designs, and short-term follow-ups. This systematic review provides such gaps by synthesizing the evidence of the influence of KD on metabolic, endocrine, and reproductive outcomes relative to other diets, safety, adherence, and best practices to guide evidence-based clinical practice.

## Review

Study design

This systematic review was conducted based on the guidelines of the Preferred Reporting Items of Systematic Reviews and Meta-Analyses (PRISMA) 2020 [16] in order to offer the required methodological rigor and clarity of reporting. To enhance the transparency level and minimize bias, prior to the commencement of the review, the study protocol was registered in PROSPERO (CRD420251146771). The research question was designed in a manner that would critically evaluate the effects of KD on metabolic, endocrine, and reproductive outcomes among overweight or obese women with polycystic ovary syndrome (PCOS).

The selection of studies was done using pre-defined criteria according to the PICOS (Population, Intervention, Comparator, Outcomes, Study design) framework [17]. The inclusion criteria were adult women aged 18 years or older and confirmed to have PCOS based on either the Rotterdam or National Institutes of Health (NIH) diagnostic criteria and classified as overweight or obese (body mass index (BMI) 25 kg/m^2^ or more, or a waist circumference greater than 80 cm). Studies were eliminated that included pregnant women, those with severe renal or hepatic impairment, or those with a bariatric surgery history because these factors alone could have a significant impact on metabolic outcomes.

The intervention of interest was a KD, which is simply described as a diet that contains 50 g or less of carbohydrates per day or less than 10% of total daily calories as carbohydrates. These involved the standard KD, the modified KD, and the cyclical KD. Research had to be done to compare the KD with an active comparator (e.g., other dietary interventions) or a passive comparator (e.g., usual dietary practices or none).

The main outcomes of interest were the measures of weight loss and body composition changes, insulin resistance measured by homeostatic model assessment of insulin resistance (HOMA-IR) [18] or fasting blood glucose and insulin, and the changes in androgen levels, such as total testosterone and SHBG. Secondary outcomes included lipid profile changes (low-density lipoprotein (LDL), high-density lipoprotein (HDL), and triglycerides), regular menstrual cycle and ovulation rates, and records of adverse effects of the dietary intervention, including keto-flu symptoms or dyslipidemia.

In terms of study design, randomized controlled trials (RCTs) were selected as the most preferable studies to include because of their strong design when it comes to determining causality. Non-RCTs and prospective cohort studies were also included (to widen the evidence base. Case reports, narrative reviews, cross-sectional studies, animal studies, and conference abstracts that lacked a full-text article were excluded to keep the review focused on higher-quality evidence.

Search strategy and information sources

The systematic and exhaustive search strategy was designed and implemented in various electronic databases to have a comprehensive coverage of the available literature. PubMed/MEDLINE, Embase, Scopus, Web of Science, and Cochrane Library were searched to find peer-reviewed articles of relevance. Additionally, ClinicalTrials.gov and the World Health Organization International Clinical Trials Registry Platform (WHO ICTRP) were searched as a source of grey literature potentially containing research that fits the inclusion criteria.

The search strategy used a concatenation of Medical Subject Headings (MeSH) terms and free-text keywords pertaining to PCOS, KD, overweight and obesity status, and outcomes of interest. As an example, in PubMed, the search query included the terms “polycystic ovary syndrome” or “PCOS”, as well as “low carbohydrate diet” or “ketogenic diet”; in addition, the terms “obesity” or “overweight” and outcome-related terms such as “insulin resistance” or “weight loss” were included. Only human studies were searched in English, considering that it was necessary to ensure that the analysis was feasible; however, no limitations regarding date were used to limit the number of studies that were retrieved.

Study selection process

The selection of the study was done in two stages to provide methodological rigor. During the first step, the titles and abstracts of all records retrieved were filtered with respect to the predefined eligibility criteria by two independent reviewers (AMD & MSH). Articles that were evidently not satisfying the inclusion criteria were eliminated at this point. Potential eligible studies were then retrieved and evaluated by the same reviewers on a more detailed basis. Any disagreements between reviewers on whether to include a study or not were resolved by discussion or consultation with a third reviewer in order to reach a consensus.

A PRISMA flow diagram was used to record the study selection process and gave a clear explanation of the number of records identified, screened, assessed as eligible, and finally incorporated in the review and why the records were excluded at each step. The method was reproducible and reduced selection bias.

Data extraction

A standardized, piloted form was used to extract data to ensure consistency and the ability to capture the relevant information of each of the included studies. The data collected covered several key areas. Study characteristics included the first author's name, year of publication, country of origin, and the study design. Participant information comprised sample size, average age, baseline body mass index (BMI), and the specific diagnostic criteria used to confirm PCOS.

Details about the intervention focused on the type of KD followed, the duration of the intervention, macronutrient composition, and any methods used to monitor dietary adherence, such as food diaries or biomarker testing. For studies with comparator groups, information about the alternative dietary intervention or standard care approach was recorded. Outcome data included measurements taken at baseline and after the intervention, covering metabolic, hormonal, reproductive, and safety parameters. Whenever multiple follow-up points were reported, data from the longest follow-up period were used to better understand the sustainability of the diet's effects. Finally, safety was assessed through reports of adverse events, as well as dropout rates and reasons for discontinuation.

Quality and risk of bias assessment

The methodological rigor and potential biases of the included studies were critically evaluated using appropriate established tools. Randomized trials were assessed using the Cochrane Risk of Bias tool version 2.0 (RoB 2.0) [19], evaluating domains such as randomization procedures, adherence to protocols, completeness of outcome data, blinding, and selective reporting. Non-randomized studies were assessed with the Risk of Bias in Non-randomized Studies - of Interventions (ROBINS)-I tool [20], which examines confounding, participant selection, intervention classification, deviations from intended interventions, missing data, outcome measurement, and reporting biases.

Data synthesis

Given the variability in study populations, KD protocols, comparison groups, outcome measures, and follow-up durations, performing a quantitative meta-analysis was not feasible due to high clinical and methodological heterogeneity. Instead, a narrative synthesis approach was adopted to systematically describe and compare studies' characteristics, results, and methodological quality.

Given the variability in study populations, KD protocols, comparison groups, outcome measures, and follow-up durations, substantial clinical and methodological heterogeneity was anticipated. Although quantitative assessment of statistical heterogeneity (e.g., I²) was not formally calculated due to the absence of a meta-analysis, the decision to forego quantitative synthesis was guided by predefined criteria: considerable heterogeneity in intervention design, outcome measurement tools, and study populations precluded meaningful pooling of effect estimates. Instead, a narrative synthesis approach was adopted to systematically describe and compare studies' characteristics, results, and methodological quality.

Results

**Figure 1:**
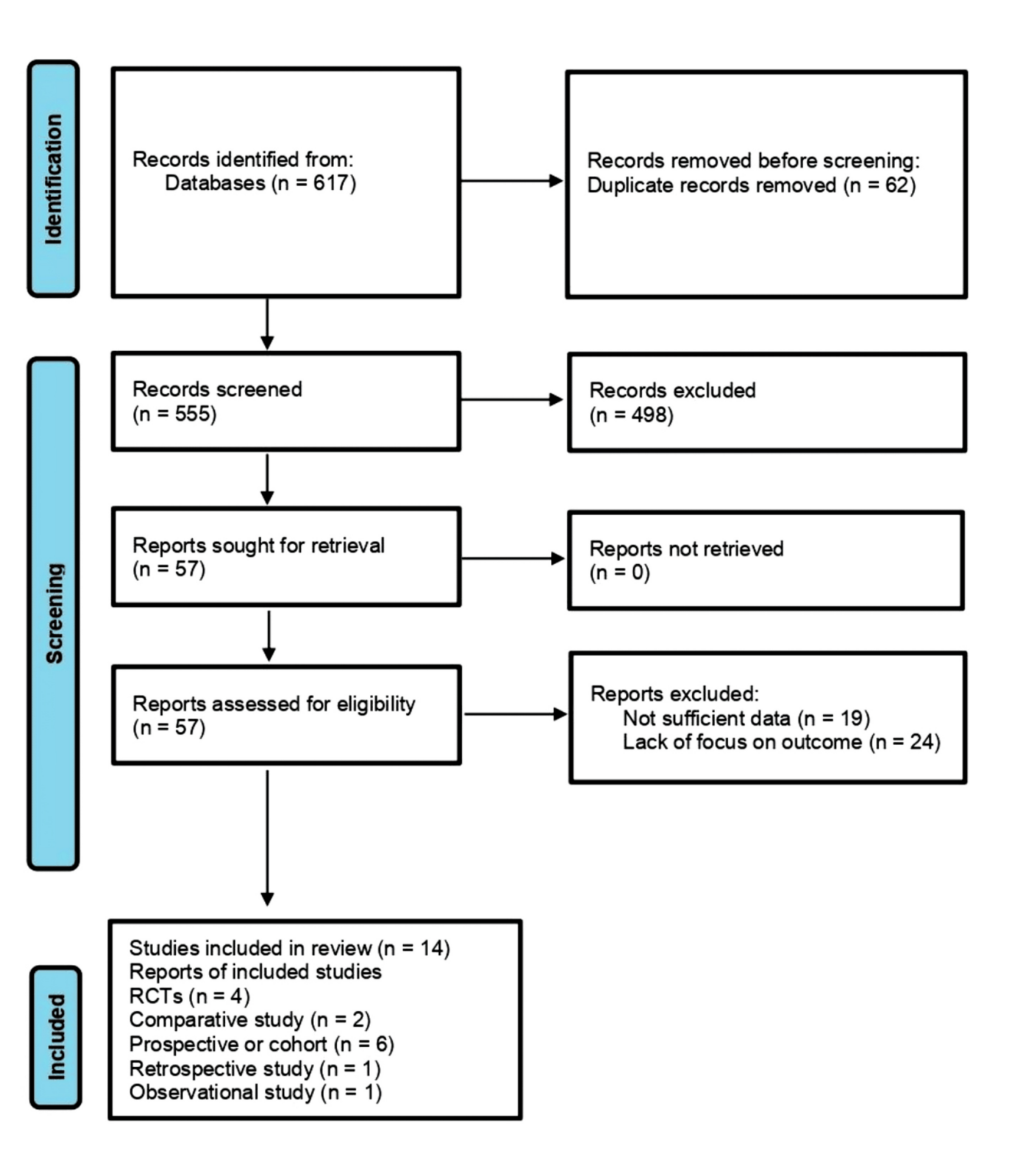
PRISMA 2020 flow diagram for the systematic review PRISMA: Preferred Reporting Items for Systematic Reviews and Meta-Analyses

The systematic review entailed an extensive review of 14 studies that explored the effects of KD on metabolic, hormonal, reproductive, and safety outcomes in overweight or obese women with PCOS. Out of 617 records identified, 62 duplicates were removed and 555 records screened. After excluding 498 records, 57 full-text articles were assessed for eligibility. Of these, 43 were excluded, leaving 14 studies [21-34] included in the final review. Figure 1 shows the PRISMA flow diagram of the screening of the included studies.

Key Characteristics of the Studies

The studies varied in design, including four RCTs, two comparative non-randomized studies, and eight prospective or retrospective cohort and single-arm interventions. Sample sizes ranged from 11 to 144 participants, primarily comprising overweight or obese women diagnosed with PCOS, mainly according to the Rotterdam or NIH criteria. The KD implemented was predominantly low in carbohydrates (<50 g per day), high in fat (60-80% of energy), with moderate protein intake, and interventions lasted between 45 days and 120 days. Some studies incorporated additional behavioral support, structured exercise programs, or carbohydrate reintroduction phases to improve adherence and outcomes. While many studies did not feature a comparator, those that did frequently used Mediterranean diet variants or standard medical/pharmacological treatments for comparison. Table 1 presents a summary of the key characteristics of the 14 studies included in this systematic review.

**Table 1:** Characteristics of the included studies BMI = body mass index; KD = ketogenic diet; VLCKD = very low-calorie ketogenic diet; LCD = low-calorie diet; MD = Mediterranean diet; PCO = polycystic ovaries; PCOS = polycystic ovary syndrome; PMCD = portfolio moderate-carbohydrate diet; DRSP/EE = drospirenone/ethinylestradiol

Studies	Country (Setting)	Study Design	Sample Size (n)	Population Characteristics	PCOS Diagnostic Criteria	Intervention (KD Type & Duration)	Comparator
Missel et al., 2021 [21]	United States	Single-arm prospective pilot study	29	Overweight/obese women, BMI 25–50 kg/m²	Rotterdam criteria (hyperandrogenism + oligomenorrhea/anovulation)	Multicomponent very-low-carbohydrate program with positive affect and mindfulness training, 16 weeks	None
Pandurevic et al., 2023 [22]	Italy	Randomized controlled trial, open-label	30 (27 completed)	Obese women with PCOS, BMI 28–40 kg/m², age 18–45 years	NIH criteria (oligo/amenorrhea + hirsutism and/or hyperandrogenemia)	VLCKD (Pronokal® method): 8 weeks VLCKD (600–800 kcal/day) + 8 weeks LCD (1050–1400 kcal/day)	Mediterranean LCD, 16 weeks (1200–1420 kcal/day; 15% protein, 30% fat, 55% carbs)
Masood et al., 2023 [23]	Pakistan	Non-randomized comparative pre–post	80 (40/group)	Premenopausal women with PCOS, age 18–45 years, BMI >30 kg/m²	Reported Rotterdam (not clearly specified)	KD: 600 kcal/day, 30 g carbs/day, 1.1–1.2 g/kg protein, 30 g fat/day	Hypocaloric Mediterranean diet (55% carbs, 25% fat, 20% protein), 9 weeks
Magagnini et al., 2022 [24]	Italy	Retrospective observational	25	Obese, non-diabetic women with PCOS, BMI 30–34.9 kg/m², mean age 25.4 ± 3.4 years	Rotterdam consensus (≥2 of 3: hyperandrogenism, ultrasound PCO, oligo/anovulation)	VLCKD, 12 weeks	None
Cincione et al., 2021 [25]	Italy	Pre–post, single-arm	17	Overweight/obese women with PCOS, BMI >25 kg/m², mean age 28.5 ± 5.4 years	Rotterdam criteria (oligo/amenorrhea + hyperandrogenism + ultrasound PCO)	Mixed ketogenic diet, 45 days	None
Paoli et al., 2020 [26]	Italy	Single-arm prospective	14	Overweight women with PCOS, BMI ≥25 kg/m², mean age 27.5 ± 8.3 years	Rotterdam criteria (≥2 of 3: oligo/amenorrhea, hyperandrogenism, ultrasound PCO)	Ketogenic Mediterranean diet with phytoextracts (KEMEPHY), 12 weeks	None
Cincione et al., 2023 [27]	Italy	Randomized controlled trial	144 (73 KD vs. 71 MD)	Overweight/obese women with PCOS, BMI 25–49.9 kg/m², age 18–45 years, treatment-naïve	ESHRE/ASRM criteria (≥2 of 3: hyperandrogenism, oligomenorrhea/amenorrhea, ultrasound PCO)	Mixed KD vs. modestly hypocaloric Mediterranean diet, 45 days	Mediterranean diet
Mavropoulos et al., 2005 [28]	United States	Single-arm pilot study	11	Women with BMI >27 kg/m² (mean 38.5), mean age 34.5 years; 80% Caucasian	Clinical diagnosis (chronic anovulation ± hyperandrogenemia)	Low-carbohydrate KD, 24 weeks	None
Li et al., 2025 [29]	China	Retrospective comparative	70 (35 KD vs. 35 comparator)	Overweight/obese women with PCOS, BMI ≥24 kg/m², mean age 31.2 ± 4.1 years	Updated Rotterdam (≥2 of 3: anovulation/amenorrhea, hyperandrogenism, ultrasound PCO)	Flexible KD, 3 months	Comprehensive intervention (lifestyle + DRSP/EE oral contraceptives)
Sharifi et al., 2024 [30]	Iran	Randomized controlled trial, open-label	46 (40 completed: 21 PMCD, 19 KD)	Overweight/obese women with PCOS, mean age 30.2 years, mean BMI 29.3 kg/m²	Rotterdam criteria	KD vs. Portfolio moderate-carbohydrate diet (PMCD), 8 weeks	PMCD
Meneghini et al., 2023 [31]	Italy (Rome, Sandro Pertini Hospital)	Non-randomized controlled	84 (42 VLCKD, 42 MD)	Overweight/obese women with PCOS, IVF candidates; mean age 33.9 years; BMI 31.2 ± 4.3 (VLCKD) vs. 28.7 ± 2.2 (MD)	Rotterdam (2003 ESHRE consensus)	VLCKD, ≥120 days	Mediterranean diet (1400 kcal/day)
Li et al., 2021 [32]	China	Randomized controlled pilot, open-label	20 (18 completed)	Obese women with PCOS + liver dysfunction, BMI 28–32, age 18–50 years; ALT/AST ≥1.5×ULN	Revised Rotterdam (≥2 of 3: anovulation, hyperandrogenism, ultrasound PCO)	KD, 12 weeks	Pharmacological treatment (Essentiale + Yasmin after liver normalization)
Yang et al., 2022 [33]	China	Prospective cohort (double-blind stratification)	55	Overweight/obese women with PCOS, BMI ≥24, age 20–40 years; stratified by uric acid (hyperuricemia n = 27, non-hyperuricemia n = 28)	Rotterdam criteria + 2018 Chinese guidelines	Flexible KD, 12 weeks	None
Rossetti et al., 2024 [34]	Italy (Rome, Sapienza University)	Single-center pilot, prospective	18 enrolled, 12 completed (33% dropout)	PCOS phenotype A, mean age 26 ± 6 years; 28% normal weight, 28% overweight, 44% obese	Rotterdam (oligomenorrhea >35 days)	KD (<50 g carbs, 1.3–1.4 g/kg protein) 45 days + carb reintroduction 45 days; 6-month follow-up	None

Table 2 summarizes the specific dietary interventions and protocols employed across the included studies. KD varied in carbohydrate allowance, typically ranging from less than 20 g to under 50 g per day, with fat contributing substantially to total energy intake - often between 60% and 75%. Protein intake was usually moderate, consistent with the Institute of Medicine guidelines, although amounts varied from about 18% to 50% of total calories or 1.1-1.4 g per kilogram body weight, depending on the study. Energy intake across studies ranged widely, from very low-calorie diets providing approximately 600 kcal/day to isocaloric or mild hypocaloric regimens tailored to individual basal metabolic rates. Intervention durations spanned between approximately 45 days and 120 days, with one study including an additional carbohydrate reintroduction phase and follow-up period. Most studies incorporated various levels of behavioral or clinical support, such as dietitian supervision, mindfulness and positive affect training, exercise protocols, supplementation with vitamins and minerals, and the use of tracking tools or digital apps for dietary adherence. Monitoring methods frequently included self-reported surveys, urine or blood ketone measurements, bioimpedance analysis, and regular contact with study coaches or clinicians to enhance compliance.

**Table 2:** Intervention details and dietary protocol KD = ketogenic diet; VLCKD = very low-calorie ketogenic diet; LCD = low-calorie diet; MD = Mediterranean diet; PMCD = portfolio moderate-carbohydrate diet; KEMEPHY = ketogenic Mediterranean diet with phytoextracts; LCKD = low-carbohydrate ketogenic diet; IOM = Institute of Medicine; kcal = kilocalorie; BHB = β-hydroxybutyrate; MCT = medium-chain triglyceride

Studies	Diet Type	Carbohydrate Allowance	Fat	Protein	Energy Intake	Duration	Protocol and Support	Monitoring
Missel et al., 2021 [21]	Very-low-carbohydrate (VLC) multicomponent program	20–35 g/day	High (implied)	Moderate (per IOM guidelines)	Not specified	16 weeks	Online modules, positive affect training, mindfulness, MyFitnessPal tracking, diet coach contact, text messages, urine ketone strips	Weekly self-report surveys; urinary ketone testing (25/29 positive); diet coach monitoring
Pandurevic et al., 2023 [22]	VLCKD (Pronokal® method)	<50 g/day (vegetables)	Not specified	High-biological-value protein (milk, soy, eggs, peas, cereals)	600–800 kcal/day (VLCKD); 1050–1400 kcal/day (LCD)	16 weeks (8 VLCKD + 8 LCD)	Vitamin/mineral supplements (K, Na, Mg, Ca, omega-3); exercise protocols; dietitian supervision	Weekly urine ketone testing (Ketostix)
Masood et al., 2023 [23]	Mixed ketogenic diet	≤30 g/day	High fat (implied)	1.1–1.2 g/kg/day; whey protein + animal proteins	~600 kcal/day	9 weeks	VLCKD vs Mediterranean diet (55% carbs, 25% fat, 20% protein); Mediterranean diet individualized	Supplementation (K, Mg); 2 L water/day; adherence calls biweekly
Magagnini et al., 2022 [24]	VLCKD (three-phase)	<30 g/day (~13% energy)	~44%	~43% (1.2–1.5 g/kg)	600–800 kcal/day (active); 1200–1500 (re-education); 1500–2000 (maintenance)	12 weeks	Protein preparations (milk, peas, whey); vitamin/mineral/omega-3 supplements	Weekly β-hydroxybutyrate (target 0.5–0.7 mmol/L)
Cincione et al., 2021 [25]	Mixed VLCKD	≤30 g/day (10–20 g/day average)	~30 g/day (mainly olive oil, nuts, fish)	35–40% daily calories (1.1–1.2 g/kg)	~600 kcal/day	45 days	Whey protein (breakfast/lunch), nuts (snack), meat/fish + vegetables + olive oil (dinner); multivitamins; ≥2 L water/day	Daily blood ketones (Glucomen LX); urine ketones (Ketostix); bioimpedance
Paoli et al., 2020 [26]	KEMEPHY (ketogenic Mediterranean diet with phytoextracts)	20.3 ± 5.2 g/day (4.8% energy)	71.1 ± 9.3% (132 g/day)	24.1 ± 5.6% (101 g/day; 1.23 g/kg)	~1600–1700 kcal/day	12 weeks	Mediterranean-style foods + supplements; herbal extracts	Weekly blood β-hydroxybutyrate (target >0.5 mmol/L)
Cincione et al., 2022 [27]	Mixed KD vs Mediterranean diet	KD: ≤30 g/day; MD: ~55% carbs	KD: ~30 g/day; MD: ~25%	KD: 1.1–1.2 g/kg; MD: ~20%	KD: ~600 kcal/day; MD: −500 kcal/day from baseline	45 days	KD: whey protein + multivitamins; MD: Mediterranean pyramid foods	KD: daily ketone monitoring; MD: nutritionist counseling
Mavropoulos et al., 2005 [28]	LCKD	<20 g/day	Unlimited animal foods, limited cheese, unlimited eggs	Not specified	Ad libitum (animal foods)	24 weeks	Biweekly group meetings, counseling, medication adjustment	Urinary ketones, food records
Li et al., 2025 [29]	Flexible KD	3–5% energy (≤50 g/day)	70–75%	20–27%	≥ basal metabolic rate	3 months	Gradual macronutrient adjustment; personalized meal plans; resistance exercise	Daily urine/weight logs; weekly blood ketones; biweekly physician visits
Sharifi et al., 2024 [30]	KD vs PMCD	KD: <30 g/day (10% energy); PMCD: 40% carbs	KD: 70%; PMCD: 40%	Both ~20%	500–700 kcal deficit	8 weeks	KD: animal proteins, olive oil, low-carb vegetables; PMCD: plant-based cholesterol-lowering foods	Urinary ketones (KD); 3-day food logs; adherence monitoring
Meneghini et al., 2023 [31]	VLCKD vs Mediterranean diet	VLCKD: 25 g/day (20% carbs); MD: 55% carbs	VLCKD: 30% fat; MD: 30% fat	VLCKD: 50% protein; MD: 15% protein	VLCKD: 800–1400 kcal/day; MD: 1400 kcal/day	120 days	VLCKD with MCT oil, supplements; MD: traditional Mediterranean	VLCKD: urinary ketones; MD: not specified
Li et al., 2021 [32]	KD	≤50 g/day (5–10% energy)	70–75%	18–27%	1300–1500 kcal/day	12 weeks	Dietitian menus, wax gourd/konjac allowed, resistance exercise	Daily urine ketones; phone follow-up
Yang et al., 2022 [33]	Flexible KD	<50 g/day (5–10% energy)	70–75%	18–27%	Personalized (basal metabolism)	12 weeks	Gradual fat increase; dietitian-guided; daily consultation	Daily serum ketones; bioimpedance; safety checks
Rossetti et al., 2024 [34]	KD with carb reintroduction	<50 g/day (KD phase); gradual reintroduction	High fat (remaining calories)	1.3–1.4 g/kg	Isocaloric (normal weight) or mild hypocaloric (overweight)	90 days (45 KD + 45 reintroduction) + 6-month follow-up	No meal replacements; vitamin/mineral supplements; 2 L water/day	Capillary β-hydroxybutyrate every 3 weeks

Outcome Measures and Assessment Methods

Table 3 provides results in the anthropometry, metabolic, hormonal, and reproductive realms. Body weight, BMI, waist/hip circumference, and fat mass (DEXA or BIA) were measured in most of the studies. Biochemical measurements involved fasting glucose, insulin, HOMA-IR, HbA1c, lipids, liver enzymes, and ovarian markers (anti-Mullerian hormone (AMH), luteinizing hormone (LH), follicle-stimulating hormone (FSH), SHBG, estradiol, progesterone). Reproductive outcomes were menstrual regularity, ovulation monitoring, IVF-related parameters, and pregnancy outcomes. Safety outcomes were documented as adverse events and dropout, and compliance with safety measures, and ketosis in most studies was confirmed through β-hydroxybutyrate tests or urinary strips.

**Table 3:** Outcomes measured across studies BMI = body mass index; WC = waist circumference; HC = hip circumference; WHR = waist-to-hip ratio; FM = fat mass; FFM = fat-free mass; TBW = total body water; BMR = basal metabolic rate; FBM = fat body mass; LBM = lean body mass; VAT = visceral adipose tissue; BF = body fat; BFP = body fat percentage; VFA = visceral fat area; RMR = resting metabolic rate; LAP = lipid accumulation product; VAI = visceral adiposity index; AFC = antral follicle count; AMH = anti-Müllerian hormone; DHEAS = dehydroepiandrosterone sulfate; 17-OHP = 17-hydroxyprogesterone; SHBG = sex hormone-binding globulin; HbA1c = glycated hemoglobin; HOMA = homeostatic model assessment; CMP = comprehensive metabolic panel; BUN = blood urea nitrogen; CBC = complete blood count; CLIA = chemiluminescence immunoassay; ECLIA = electrochemiluminescence immunoassay; PCOS-Q = polycystic ovary syndrome questionnaire; PROMIS = Patient-Reported Outcomes Measurement Information System

Studies	Anthropometric Outcomes	Metabolic Outcomes	Endocrine Outcomes	Reproductive Outcomes	Lipid Outcomes	Safety/Adverse Events	Methods/Assessments
Missel et al., 2021 [21]	% Weight change, BMI, weight (kg)	HbA1c, glucose, insulin, HOMA2-IR	SHBG, testosterone	Menstrual predictability, PCOS-QOL (multiple domains), menstrual symptoms, emotions, infertility, weight concerns, body hair	LDL, HDL, triglycerides	Kidney stones (n = 2), cramping, blurry vision, headaches, sleep issues	Clinical measurements, blood biochemistry, PCOSQ, PROMIS, remote scale
Pandurevic et al., 2023 [22]	BMI, weight, WC, HC, BIA fat mass, fat-free mass, BP, HR	Fasting glucose/insulin, HOMA-IR, cholesterol, HDL, LDL, triglycerides, AST, ALT	Testosterone, SHBG, free testosterone, androgens (LC-MS/MS), LH, FSH, estradiol	Ovulation monitoring (ultrasound + hormones), ovarian volume, stroma/total area	Total cholesterol, HDL, LDL, triglycerides	Kidney stones, GI intolerance, dropouts, compliance issues	BIA, LC-MS/MS, ultrasound, videodermoscopy, psychological questionnaires
Masood et al., 2023 [23]	Weight, BMI, WC, HC, WHR, energy intake	Glucose, insulin, HOMA-IR, C-peptide, HbA1c, albumin	LH, FSH, LH/FSH, free and total testosterone, SHBG	Not assessed	Cholesterol, HDL, LDL, triglycerides	Not assessed	Blood analysis, anthropometry, SPSS analysis
Magagnini et al., 2022 [24]	Weight, BMI, WC, anthropometric parameters	HOMA index, insulin resistance	SHBG, AMH, progesterone (day 21), luteal markers	Ovulatory function (progesterone >15.9 ng/mL)	Not reported	No adverse events, no dropouts	Cobas Elecsys assays, BIA
Cincione et al., 2021 [25]	Weight, BMI, WC, HC, WHR, FM, FFM, MM, TBW, BMR	Glucose, insulin, C-peptide, HOMA, albumin	LH, FSH, LH/FSH, testosterone, free testosterone, SHBG	Menstrual cycle regularity, gynecological evaluation, pregnancy outcomes	Comprehensive lipid profile	Daily ketone monitoring, clinical outcomes	BIA (Tanita TBF-300A), immunochemiluminescence, enzymatic methods, RIA
Paoli et al., 2020 [26]	Weight, BMI, FBM, LBM, FBM%, LBM%, VAT, WC	Glucose, insulin, HOMA-IR, metabolic flexibility	LH, FSH, LH/FSH, testosterone, free testosterone, SHBG, estradiol, progesterone, DHEAS	Not assessed	Lipid profile (cholesterol, HDL, LDL, triglycerides)	Hirsutism (Ferriman–Gallwey score)	DEXA, immunochemiluminescence, enzymatic assays, RIA
Cincione et al., 2022 [27]	Weight, BMI, WC, HC, WHR, FM, FFM, TBW, BMR	Glucose, insulin, C-peptide, HOMA-IR, albumin	LH, FSH, LH/FSH, testosterone, free testosterone, SHBG	Menstrual cycle and gynecological evaluation	Not reported	Not reported	BIA, immunochemiluminescence, RIA, anthropometry
Mavropoulos et al., 2005 [28]	Weight, BMI	Fasting insulin, glucose, HbA1c	Testosterone, free testosterone, % free testosterone, LH/FSH	Fertility (pregnancy), PCOS-specific questionnaire (domains: emotions, hair, weight, infertility, menstruation)	Cholesterol, HDL, LDL, triglycerides	Blood pressure, dropouts	Tanita scale, BP cuff, immunoassay, ultrafiltration, PCOS-Q
Li et al., 2025 [29]	Weight, BMI, body composition	FBG, insulin, HOMA-IR	FSH, LH, LH/FSH, estradiol, testosterone	Not assessed	Cholesterol, HDL, LDL, triglycerides	ALT, AST, BUN, creatinine	Electrochemiluminescence, enzyme assays, body composition, metabolic rate
Sharifi et al., 2024 [30]	Weight, BMI, WC, HC, FBM, LBM, TBW, TB protein/mineral, RMR	Glucose, insulin, HOMA-IR, HOMA-B	LH, FSH, free testosterone, DHEA-S	Menstrual cycles, pregnancies (n = 2, KD group)	Cholesterol, HDL, LDL, triglycerides, LAP, VAI	Dropouts, adherence	BIA, biochemistry, ELISA, urinary ketones, IPAQ-SF
Meneghini et al., 2023 [31]	BMI, circumferences (waist, hip, thigh), WHR	HOMA, cholesterol, HDL, triglycerides	FSH, LH, FSH/LH, estradiol, AMH, 17-OHP, androstenedione, testosterone	Menstrual regularity, AFC, OHSS risk during IVF	Cholesterol, HDL, triglycerides	No adverse events	Ultrasound, CLIA, ECLIA, anthropometry
Li et al., 2021 [32]	Weight, BMI, BF, BFP, VFA, WHR	Glucose, liver function markers (primary)	FSH, LH, estradiol, testosterone, progesterone, prolactin	Menstrual length and regulation	Cholesterol, HDL, LDL, triglycerides	ALT, AST (primary), liver US grading, pelvic US	Inbody 770 BIA, transvaginal US, standardized bloods
Yang et al., 2022 [33]	Weight, BMI, BF%, FM, LBM, muscle mass, VAT, metabolism, WHR	Glucose, TGs, HDL, LDL, cholesterol, APOA, APOB, uric acid (primary)	Testosterone, FSH, LH, LH/FSH, estradiol, progesterone	Menstrual resumption (hyperuricemia vs non-hyperuricemia)	Comprehensive lipid profile	ALT, AST, albumin, creatinine, BUN, Hb (safety)	BIA, enzymatic methods, immunoassays
Rossetti et al., 2024 [34]	Weight, BMI, WC, HC, DEXA body composition (android/gynoid, VAT mass/volume/area, FFM)	Glucose, insulin, HOMA-IR, CMP (urea, creatinine, proteins, albumin, electrolytes)	Estradiol, FSH, LH, progesterone, testosterone, TSH, FT3, FT4, cortisol	Menstrual cycle frequency (primary), ovarian morphology (3D US), hirsutism (FG score)	Cholesterol, HDL, LDL, triglycerides	CBC, liver (AST, ALT, GGT), renal function, adverse events, dropouts	DEXA, 3D US, capillary BHB monitoring, biochemical assays, safety questionnaires

Effects of KD on Weight and Body Composition

The overall and important finding was that weight loss was consistent and statistically significant across studies (Table 4). The mean reduction was 7.67% reported by Missel et al. [21] in 16 weeks, and 18 ± 4.2 kg in 12 weeks reported by Magagnini et al. [24]. The preferential fat mass loss rate of 9.4 kg in 45 days was attained by Cincione et al. [27], and 12.1% weight loss at 24 weeks was observed by Mavropoulos et al. [28]. RCTs showed better anthropometric results in VLCKD than in Mediterranean controls [22,27]. Meneghini et al. [31] and Li et al. [29] validated higher BMI, waist, and fat mass decreases in ketogenic groups. The visceral adiposity and fat mass had improved significantly [26,34], which highlights the importance of central fat reduction as one of the most important KD effects.

**Table 4:** Key results summary BMI = body mass index; WC = waist circumference; HC = hip circumference; FM = fat mass; FFM = fat-free mass; BF = body fat; BFP = body fat percentage; VFA = visceral fat area; VAT = visceral adipose tissue; WHR = waist-to-hip ratio; SHBG = sex hormone-binding globulin; AMH = anti-Müllerian hormone; LH = luteinizing hormone; FSH = follicle-stimulating hormone; DHEAS = dehydroepiandrosterone sulfate; HbA1c = glycated hemoglobin; HOMA-IR = homeostatic model assessment of insulin resistance; TG = triglycerides; HDL = high-density lipoprotein cholesterol; LDL = low-density lipoprotein cholesterol; ALT = alanine aminotransferase; AST = aspartate aminotransferase; BHB = β-hydroxybutyrate; AFC = antral follicle count; PCOS-QOL = polycystic ovary syndrome quality of life questionnaire; VLCKD = very low-calorie ketogenic diet; KD = ketogenic diet; MD = Mediterranean diet; PMCD = portfolio moderate-carbohydrate diet; BP = blood pressure; ns = nonsignificant

Studies	Anthropometric Results	Metabolic Results	Endocrine Results	Reproductive Results	Lipid Results	Safety/Adverse Events
Missel et al., 2021 [21]	Mean weight loss 7.67% (SD = 6.10, p < 0.001); BMI ↓2.64 kg/m² (SD = 2.14, p < 0.001); 52% achieved ≥5% weight loss	HbA1c ↓0.21% (SD = 0.27, p = 0.001); improved glucose control	SHBG ↑9.24 nmol/L (SD = 16.34, p = 0.01); testosterone trend (p = 0.09)	Improved menstrual predictability (Δ2.10, SD = 2.76, p = 0.002); better PCOS-QOL (body hair, emotions, weight concerns, infertility)	LDL ↑0.23 mmol/L (SD = 0.49, p = 0.04)	Kidney stones (n = 2), cramps/blurred vision (n = 1), headaches, sleep issues; 27% dropout; high satisfaction (median = 7/7)
Pandurevic et al., 2023 [22]	BMI ↓13.7% (VLCKD) vs ↓5.1% (control), p = 0.0003; WC ↓11.4% vs ↓2.9%, p = 0.0008; fat mass ↓24.0% vs ↓8.1%, p = 0.018; systolic BP ↓ only in VLCKD (p = 0.032)	HOMA-IR ↓23% (p = 0.024) in VLCKD; insulin ↓ (p = 0.013); cholesterol ↓ (p < 0.001)	Free testosterone ↓30.4% vs ↓12.6% (p = 0.001); SHBG ↑ in VLCKD (p < 0.001)	Ovulation improved: VLCKD 84.6% vs 35.7% (p = 0.018)	Cholesterol ↓, HDL ↓, TG ↓ (trend, p = 0.08); AST ↓ (trend, p = 0.053)	3 dropouts: 2 VLCKD (kidney stones, GI intolerance), 1 control (noncompliance); good tolerance; ketosis confirmed
Masood et al., 2023 [23]	KD: weight ↓10.9 kg vs ↓5.1 kg (p < 0.001); BMI ↓3.3 vs ↓0.4; WC ↓11.2 cm vs ↓2.6 cm	Glucose ↓12.1 vs ↓5.1; insulin ↓16.1 vs ↓6.2; HOMA-IR ↓5.2 vs ↓2.0	LH ↓5.3 vs ↓3.7; LH/FSH ratio improved; SHBG ↑19.3 vs ↑11.8; free testosterone ↓0.32 vs ↓0.15	Not assessed	TG, cholesterol, LDL ↓; HDL ↑ (all significant in KD; modest in Mediterranean)	No adverse events reported
Magagnini et al., 2022 [24]	Mean weight loss 18 ± 4.2 kg; BMI decreased; 76% shifted from obese to overweight; WC ↓ significantly (p < 0.05)	HOMA normalized (<2.5 in 96% of patients)	AMH ↓7.8 ± 3.0 ng/mL (p < 0.05); SHBG ↑ (p < 0.05); progesterone ↑ (p < 0.05)	100% achieved ovulation (progesterone >15.9 ng/mL)	Not reported	100% completion, no adverse events
Cincione et al., 2021 [25]	Weight ↓9.4 kg (p < 0.001); BMI ↓3.6 kg/m² (p < 0.001); WC ↓9.4 cm (p < 0.001); FM ↓7.9 kg (p < 0.001); small FFM ↓1.4 kg (p < 0.05)	Glucose ↓10 mg/dL (p < 0.001); insulin ↓12.9 μU/mL (p < 0.001); HOMA-IR ↓3.45 (p < 0.001); C-peptide ↓0.87 ng/mL (p < 0.001); albumin ↑ (p < 0.001)	LH ↓4.6 mIU/mL (p < 0.001); FSH ↑1.5 mIU/mL (p < 0.05); LH/FSH normalized; testosterone ↓ (p < 0.001); SHBG ↑12.4 nmol/L (p < 0.001)	100% cycle normalization; 5/5 amenorrheic regained menses; 5/12 pregnancies achieved naturally	TG ↓70 mg/dL (p < 0.001); cholesterol ↓40 mg/dL (p < 0.001); LDL ↓35 mg/dL (p < 0.001); HDL ↑15 mg/dL (p < 0.01)	Excellent compliance; ketosis maintained; no adverse events
Paoli et al., 2020 [26]	Weight ↓9.4 kg (p < 0.001); BMI ↓3.4 (p < 0.001); FBM ↓8.3 kg (p < 0.001); VAT ↓640 g (p < 0.001); WC ↓4 cm (p = 0.002)	Glucose, insulin, HOMA-IR improved (all p < 0.001)	LH/FSH normalized; testosterone, free testosterone, DHEAS ↓; estradiol, progesterone ↑; SHBG ↑ (all p < 0.05)	Not assessed	TG, cholesterol, LDL ↓; HDL ↑ (all p < 0.05)	2 withdrawals; mild hirsutism score decrease (ns); no serious events
Cincione et al., 2022 [27]	KD superior to MD: weight ↓11.4 vs ↓3.1 kg (p < 0.001); BMI ↓4.2 vs ↓1.2 (p < 0.001); WC ↓10 vs ↓2.8 cm (p < 0.001)	KD: glucose ↓13.2 vs ↓4.5 (p < 0.001); insulin ↓21.6 vs ↓6.9 (p < 0.001); HOMA-IR ↓5.7 vs ↓1.9 (p < 0.001)	KD: LH ↓5.5 vs ↓3.1; testosterone ↓7.4 vs ↓5.3; SHBG ↑18.1 vs ↑9.1; all p < 0.01	KD: 100% amenorrheic regained cycles; MD: 0% (p < 0.001)	Not reported	16 dropouts; BMI-independent effects confirmed
Mavropoulos et al., 2005 [28]	Weight ↓12.1% (p = 0.006); BMI ↓4.0 kg/m²	Insulin ↓53.7% (p = 0.002); glucose, HbA1c trends (ns)	Free testosterone ↓30% (p = 0.04); LH/FSH ↓36% (p = 0.03)	2 pregnancies achieved; QOL domain improvements (p = 0.06)	Lipids: nonsignificant changes; BP ↓	54% dropout; no adverse effects
Li et al., 2025 [29]	KD: weight ↓15.5 kg vs ↓10.0 kg (p < 0.05); BMI ↓5.9 vs ↓3.8 (p < 0.05)	KD: FBG ↓0.5 mmol/L (p < 0.001); insulin ↓4.4 mIU/L (p = 0.004); HOMA-IR ↓1.3 (p < 0.001); greater FBG reduction than control (p = 0.026)	KD: LH ↓3.4 IU/L (p < 0.001); testosterone ↓0.12 pg/mL (p = 0.001); both groups improved hormones	Not assessed	KD: TG ↓0.47 mmol/L (p = 0.004); control: HDL ↑0.36 mmol/L (p < 0.001); group differences significant	KD: 2 constipation, 1 hypoglycemia (resolved); no events in control
Sharifi et al., 2024 [30]	KD vs PMCD: weight ↓5.6 vs ↓4.4 kg; BMI ↓2.7 vs ↓1.7; FBM ↓4.4 vs ↓3.1 (all p < 0.05)	KD vs PMCD: glucose ↓8.8 vs ↓5.0; insulin ↓13.4 vs ↓7.4; HOMA-IR ↓3.5 vs ↓2.0 (all p < 0.05)	LH ↓4.4 vs ↓2.5; DHEA-S ↓0.4 vs ↓0.2; testosterone improved in both (p < 0.05)	Ferriman–Gallwey: no significant changes	PMCD superior lipid improvements; LDL ↓34 vs ↓23; HDL ↑15 vs ↑11; TG ↓58 vs ↓38	2 pregnancies (KD); 2 PMCD, 4 KD dropouts; no adverse events
Meneghini et al., 2023 [31]	VLCKD greater weight/BMI/WC/HC reduction than MD at 90 and 120 days (all p < 0.001)	HOMA index ↓1.5 vs ↓0.3 at 90 days (p < 0.001); cholesterol ↓ more in VLCKD; HDL ↑ more at 120d	VLCKD greater reductions in testosterone, AMH, androstenedione (p < 0.05)	VLCKD: more AFC ↓; more menstrual regulation (71% vs 26% at 120d, p < 0.001)	TG ↓ more in VLCKD (p < 0.05)	No adverse events; no dropouts
Li et al., 2021 [32]	KD: BMI ↓4.5 (p < 0.05); weight ↓12 kg (p < 0.05); BF, BFP, VFA ↓ significantly; WHR ↓ (p < 0.05); control: no significant changes	KD: glucose ↓0.8 mmol/L (p < 0.05); superior liver enzyme improvement vs control	Both groups: estradiol, progesterone ↓ (p < 0.05); testosterone trend	Both groups: cycle shortening (p < 0.05); KD cycles normalized faster	No lipid changes in either group	KD: ALT, AST ↓ more (p < 0.05); fatty liver resolved in 86% vs 10%; no adverse events
Yang et al., 2022 [33]	Greater weight loss in the non-hyperuricemia group (14.7 vs 11.2 kg, p < 0.05); BMI and fat % ↓ in both groups	TG ↓ significantly in both groups; glucose ↓ in both	No significant hormonal changes	Menstrual resumption: hyperuricemia 67%, non-hyperuricemia 54%	No group differences in final lipid outcomes; uric acid fluctuated	ALT, AST ↓ in both groups (p < 0.01); no gout symptoms
Rossetti et al., 2024 [34]	Weight ↓11.5% (p = 0.003); BMI ↓10.8% (p = 0.008); FM ↓18.3% (p = 0.003); VAT ↓29.7% (p = 0.037); WC ↓13.5% (p = 0.001)	Insulin ↓37.7% (p = 0.033); HOMA-IR trend (p = 0.076); AST ↓19.5% (p = 0.006); ALT ↓38.2% (p = 0.003)	FSH ↓30% (p = 0.05); LH ↓69% (p = 0.037); progesterone ↑330% (p = 0.017); testosterone ↓30% (p = 0.048)	100% achieved cycle normalization (p = 0.012); ovarian volume ↓28% (p = 0.029); hirsutism improved (p = 0.037)	HDL ↑16.6% (p = 0.004); TG ↓19.5% (p = 0.022)	33% dropout (n = 6); mild constipation; ketosis achieved (BHB >0.5 mmol/L); no serious events

Metabolic Effects: Insulin Sensitivity and Glycemic Control

Across studies, fasting insulin, glucose, and HOMA-IR improved consistently. Missel et al. [21] reduced HbA1c by 0.21% (p = 0.001). Cincione et al. [25] demonstrated rapid glucose and insulin declines in 45 days, and Magagnini et al. [24] normalized the HOMA index in 96% of participants. Pandurevic et al. [22] and Cincione et al. [27] found a greater improvement in metabolism compared to Mediterranean diets despite BMI correction. Mavropoulos et al. [28] decreased insulin by 53.7%. Li et al. [32] demonstrated significant reductions in ALT/AST in PCOS with liver dysfunction, and Li et al. [32] demonstrated better fasting glucose and HOMA-IR with KD than the comprehensive interventions. Meneghini et al. [31] have reported long-term gains at 90 and 120 days, and Sharifi et al. [30] showed a better increase in insulin sensitivity with KD compared to a moderate-carbohydrate diet.

Hormonal Modulations

KD consistently lowered androgen levels and normalized ovulatory hormones (Table 4). Missel et al. [21] observed SHBG increases (+9.24 nmol/L, p = 0.01). Pandurevic et al. [22] reported free testosterone reduction (-30.4%) with SHBG elevation. Cincione et al. [25] documented comprehensive improvements, including LH decrease, FSH rise, and improved SHBG, and Magagnini et al. [24] found AMH decreases and progesterone increases predictive of ovulation. Paoli et al. [26] and Meneghini et al. [31] showed reversal of LH/FSH ratios and significant androgen declines. Rossetti et al. [34] demonstrated dramatic LH and progesterone shifts independent of weight loss. Mavropoulos et al. [28] confirmed reductions in free testosterone and LH/FSH ratios, while Li et al. [29] found comparable hormonal benefits between KD and comprehensive interventions.

Reproductive Outcomes

Improved menstrual regularity, ovulation, and pregnancy rates were consistent findings. Pandurevic et al. [22] documented ovulation increases from 38.5% to 84.6%, Cincione et al. [25] achieved 100% menstrual improvement with natural pregnancies in 42% of participants, and Magagnini et al. [24] restored ovulation in all participants. Rossetti et al. [34] showed menstrual normalization and ovarian volume reduction in normal-weight women, indicating weight-independent effects. Meneghini et al. [31] uniquely improved IVF outcomes and reduced OHSS risk, while Sharifi et al. [30] reported pregnancies after only eight weeks. Mavropoulos et al. [28] documented pregnancies in two of five completers, reinforcing KD's reproductive potential.

Lipid Profile Changes

Effects on lipid profiles were mostly favorable but varied. Cincione et al. [25] and Paoli et al. [26] reported large triglyceride and LDL reductions with HDL increases, while Rossetti et al. [34] documented liver enzyme improvements alongside lipid gains. Sharifi et al. [30] found that a moderate-carbohydrate plant-based diet improved LDL and HDL more than KD, suggesting composition-specific cardiovascular effects. These findings highlight the need for individualized cardiovascular risk assessment and lipid monitoring in women with PCOS undergoing KD. Overall, no study showed clinically significant lipid deterioration during follow-up.

Safety and Adherence

Safety profiles ranged from mild, manageable symptoms to more concerning events. The studies by Missel et al. [21] and Masood et al. [23] included kidney stones and muscle cramps and did not report safety, respectively, though extreme caloric restriction was administered. Conversely, Magagnini et al. [24], Cincione et al. [25], and Meneghini et al. [31] reported 100% completion without adverse events. Li et al. [32] and Li et al. [29] confirmed tolerability in higher-risk groups, and Rossetti et al. [34] reported mild constipation with clear dropout reasons. Sharifi et al. [30] demonstrated good adherence and increased dropouts in KD because of non-response and pregnancy. Interventions that were shorter with organized phases of reintroduction tended to have higher adherence rates and safety.

Risk of Bias and Methodological Considerations

**Figure 2:**
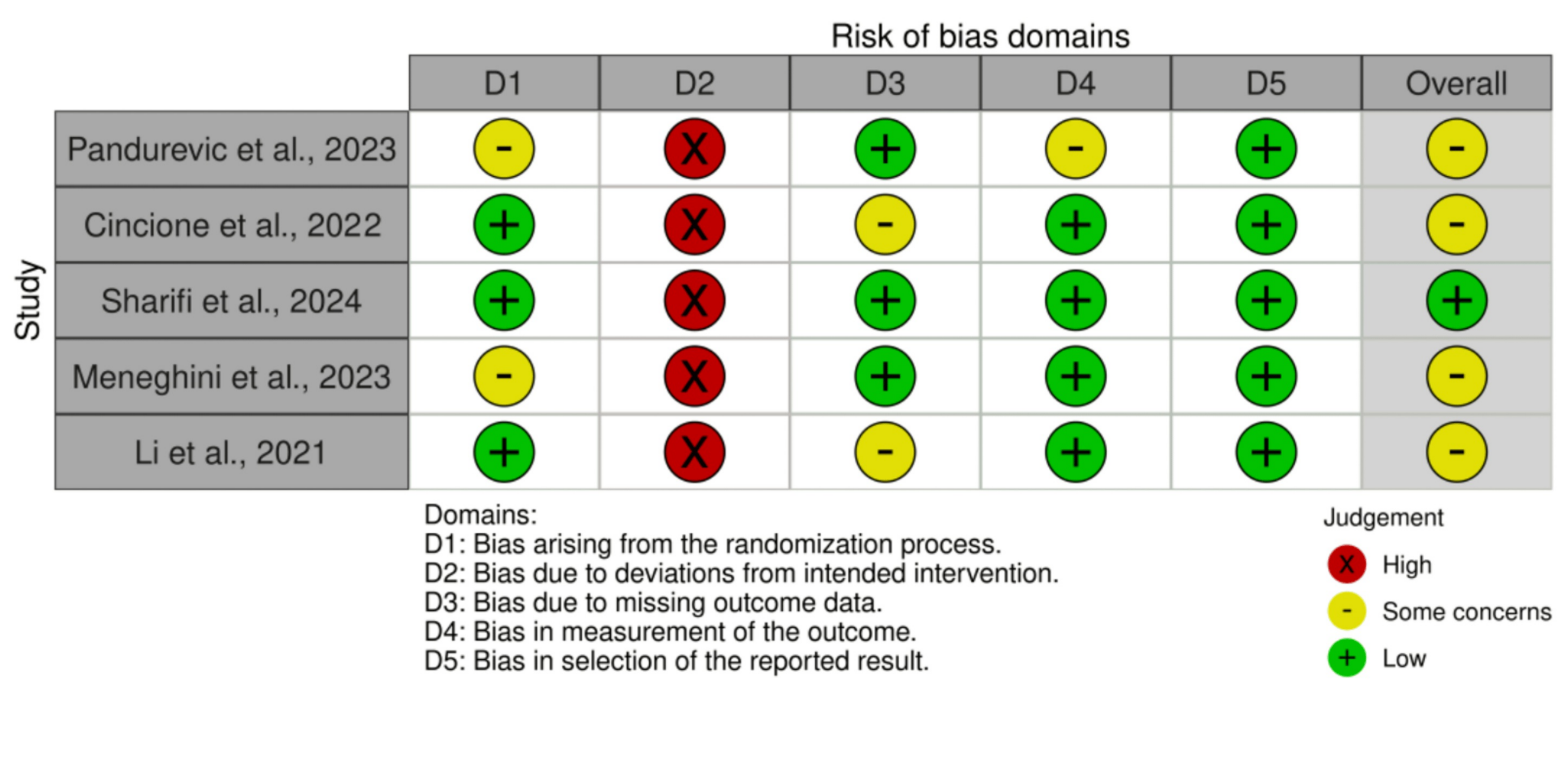
Risk of Bias Domains for Randomized Controlled Trials (Cochrane RoB 2.0)

**Figure 3:**
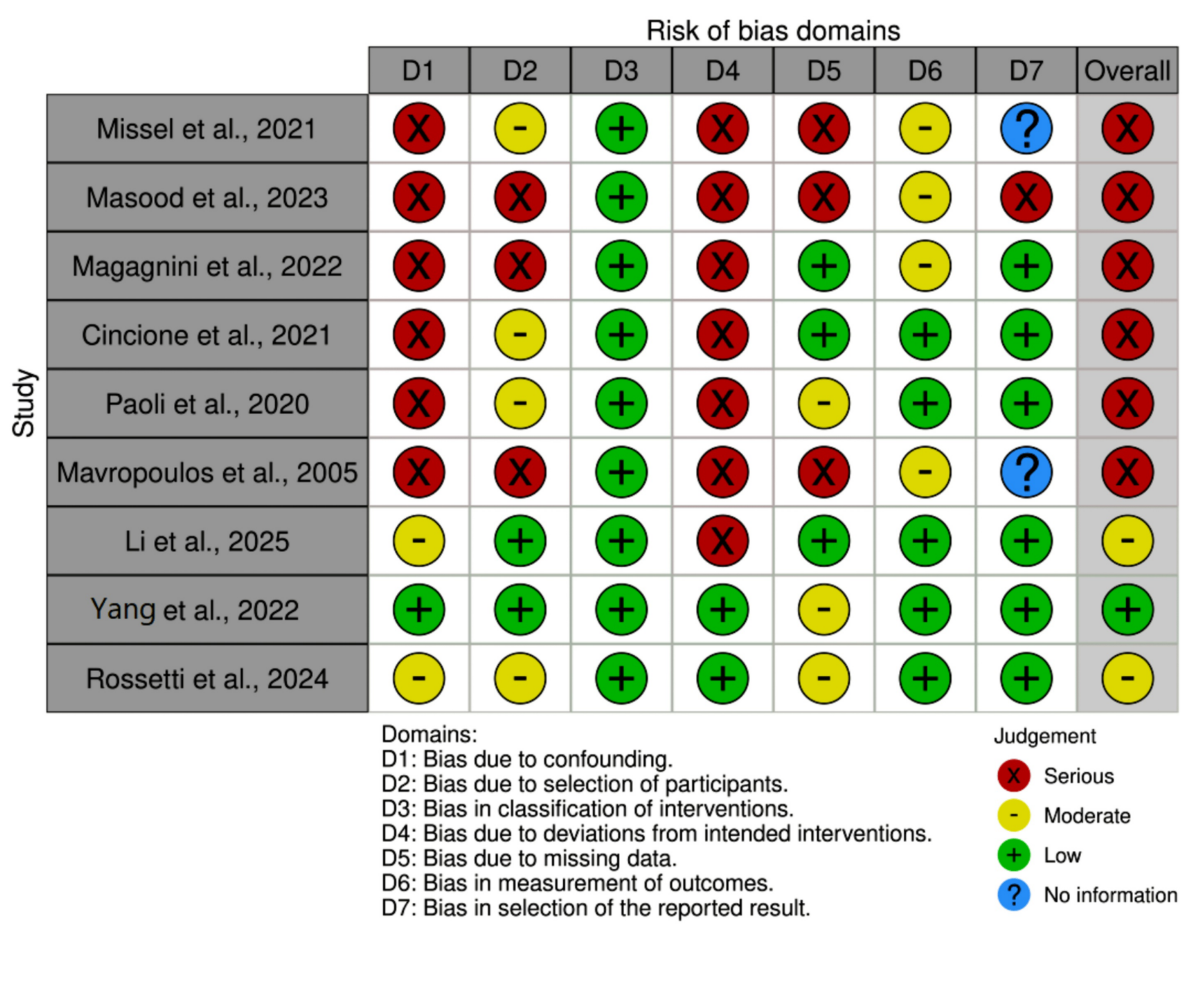
Risk of Bias Domains for Non-Randomized Studies (ROBINS-I)

The risk of bias assessment for the included RCTs using the RoB 2.0 tool is summarized in Figure 2. Across the RCTs, a consistent concern was observed regarding bias due to deviations from intended interventions (Domain 2), where all studies showed high risk, primarily due to challenges in blinding participants and personnel. Bias arising from the randomization process (Domain 1) received mixed ratings, with some studies meeting low risk and others marked as having some concerns. Outcome measurement bias (Domain 4) and bias due to missing outcome data (Domain 3) were generally rated as low risk. Despite these issues in individual domains, the overall risk of bias for most RCTs was rated as some concerns, reflecting the complexities of dietary intervention studies. For the non-randomized studies, Figure 3 illustrates the ROBINS-I results. Several domains presented serious risks of bias, notably bias due to confounding (Domain 1) and selection of participants (Domain 2), reflecting challenges inherent in observational study designs. Bias related to the classification of interventions (Domain 3) was typically low, while bias due to missing data (Domain 5) and measurement of outcomes (Domain 6) varied among studies. The selection bias of reported results (Domain 7) was unclear or moderate across most studies. Accordingly, the overall risk of bias for most non-randomized studies was serious or moderate rather than low.

Discussion

Our study examines the effects of the KD on overweight and obese women with PCOS. We also consider the effects of ketogenic nutrition on metabolic, hormonal, and reproductive outcomes, as well as patient experiences and barriers to clinical implementation. This review, through the synthesis of varied evidence, presents a better view of the therapeutic ability of KD and its limitations in the treatment of PCOS.

This systematic review examined 14 articles - RCTs, comparative cohorts, and single-arm interventions. In all of them, KD interventions were associated with clinically significant weight reduction, an average of 7-13% of baseline body weight in 8-16 weeks [22,34]. Such weight losses are clinically significant since relatively small weight changes of 5-10% have been shown to help women with PCOS to regain ovulation and insulin sensitivity [35]. The fact that KD consistently surpasses this threshold reinforces its argument as a specific intervention for weight-related complications in PCOS.

In addition to an overall loss in weight, visceral fat and central adiposity had been reduced repeatedly [24,26]. This is important in that insulin resistance, inflammation, and cardiovascular risk are closely associated with central obesity in PCOS, such that fat loss in these areas has more health benefits than weight loss per se. These results also correspond to the more general literature on low-carbohydrate diets, which emphasizes the selective mobilization of visceral fat [36].

Metabolic improvements were another consistent finding, including reductions in fasting glucose, insulin, and HOMA-IR [31,32]. These changes are meaningful because insulin resistance underpins many of the metabolic and reproductive symptoms of PCOS. Mechanistically, carbohydrate restriction reduces the post-meal insulin response and increases fat oxidation, respectively, decreasing the hormonal imbalances that are caused by hyperinsulinemia [37]. The enhancement of liver enzymes and the resolution of fatty liver in KD groups [32] should also be mentioned, as non-alcoholic fatty liver disease (NAFLD) is present in one-third of women with PCOS. The same findings using non-PCOS groups affirm the ability of KD to benefit liver fat and liver function [38], indicating a more extensive metabolic impact.

Hormonal results reported homogenous results with a decrease in total and free testosterone, and improvement in SHBG, and normalization of LH/FSH ratios [21,25]. It is of importance to reduce androgen levels since hyperandrogenism is the cause of most of the unpleasant clinical manifestations of PCOS, such as infertility, acne, and hirsutism. Some studies also reported reductions in AMH [24], suggesting improved ovarian function. This is especially relevant for women pursuing fertility treatment, as high AMH levels can signal disrupted follicular development and an increased risk of ovarian hyperstimulation syndrome (OHSS).

Reproduction, less often reported, was positive. Research reported a positive effect on ovulation, menstrual regularity, and even spontaneous pregnancy [22,25,28]. These findings are important since one of the most problematic areas for patients is reproductive dysfunction. Cincione et al. [25] reported a 42% pregnancy rate among women who regained regular cycles; however, this finding should be interpreted in the context of expected spontaneous pregnancy rates in untreated PCOS populations, which vary widely but are generally low in women with prolonged anovulation.

Rossetti et al. [34] further showed that menstrual improvements occurred even after controlling for weight loss, suggesting that ketosis may exert direct effects on ovarian physiology. If confirmed, this would broaden the relevance of KD beyond obese PCOS patients to include normal-weight women with reproductive dysfunction.

There were variable lipid profile responses. Most of the studies claimed decreased triglycerides and increased HDL cholesterol, which decreases the risk of cardiovascular disease, although LDL cholesterol increases were reported [21]. This difference shows why women with PCOS require specific monitoring, since cardiovascular risk is already increased in them. The same observations have been made in the broader literature of KD [39], highlighting the fact that lipid changes are influenced by genetics as well as diet composition.

Although these are very encouraging results, significant limitations must be admitted. The majority of the studies were small and short, 6-16 weeks, which restricted generalization and could not evaluate the long-term safety. There was a high dropout rate. For instance, the study by Mavropoulos et al. [28] had a 54% dropout rate, indicating the keto diet is very restrictive and difficult to maintain long-term. KD protocols also vary, making interpretation more difficult. Some of these used starvation-level VLCKDs (~600 kcal/day) [23,25], whereas others were more flexible or more Mediterranean-style ketogenic [26]. Such heterogeneity brings about the question of whether it is ketosis, caloric restriction, or a combination of dietary structure and behavioral support that is beneficial.

Caution and personalization are therefore needed in the clinical implementation. It has been indicated that adherence and safety can be enhanced through structured support, such as behavioral counseling [21], micronutrient supplementation, and ketone monitoring. In the absence of such support, patients can suffer in terms of dietary non-compliance to adverse events, including kidney stones, that happened in 7% of the participants in one trial. Comparative studies also emphasize tailoring dietary choices to clinical priorities. For example, KD may be superior for rapid weight loss and metabolic improvement [22,29], whereas moderate low-carbohydrate or Mediterranean diets may be safer and more sustainable for cardiovascular health [30,40].

Overall, KD demonstrates consistent short-term effects on weight loss, insulin resistance, and hormonal control in women with PCOS. Reproductive outcomes appear promising, though evidence remains less definitive. These results are in line with the previous reviews that highlight the advantages of carbohydrate restriction in PCOS [36,41]. However, dropout rates, disparate protocols, inconsistent lipid effects, and the lack of long-term cardiovascular data all suggest the importance of cautious clinical interpretation.

Future studies ought to focus on larger, well-designed RCTs using standardized KD protocols, and extend follow-ups (≥12 months) and effective monitoring of adherence and adverse events. Particular groups of people include normal-weight women [34], those with liver dysfunction [32], or women undergoing assisted reproductive procedures [31], and should be studied separately. Until they have evidence to support it, KD can be considered as a promising but experimental tool, which is most effectively applied to patient-specific objectives, risks, and preferences under the multidisciplinary supervision.

## Conclusions

This systematic review demonstrates that KD offer considerable therapeutic potential for managing metabolic, endocrine, and reproductive dysfunctions in overweight and obese women with PCOS. KD resulted in consistent clinically meaningful weight loss (7-13% in 8-16 weeks) and visceral adiposity. Metabolic changes comprised of improved insulin sensitivity, diminished fasting glucose and insulin, and improved liver enzymes. The hormonal changes included a reduction in free testosterone, elevated SHBG, and even normal LH/FSH ratios. Several studies have shown reproductive outcomes, indicating that menstrual regularity, increased ovulation rate, and spontaneous pregnancies were restored by the intervention. Although the changes in lipids were mostly positive, there was a certain inconsistency. The adverse events were generally mild, but compliance issues and other infrequent accidents, such as kidney stones, underscore the importance of surveillance in implementation. Limitations to the study include short periods (6-24 weeks), heterogeneous protocols, and inconsistent adherence levels, which restrict generalizations to long periods. KD can be discussed as an experimental technique despite its promising short-term effectiveness that should be performed individually and under the clinical guidelines. Further studies should focus on large-scale RCTs using standardized protocols and long-term follow-up to determine the long-term safety, sustainability, and the best mode of integrating into PCOS management.
